# Therapeutic properties in Tunisian hot springs: first evidence of phenolic compounds in the cyanobacterium *Leptolyngbya* sp. biomass, capsular polysaccharides and releasing polysaccharides

**DOI:** 10.1186/s12906-016-1492-3

**Published:** 2016-12-13

**Authors:** Lamia Trabelsi, Amira Mnari, Mohamed M. Abdel-Daim, Salwa Abid-Essafi, Lotfi Aleya

**Affiliations:** 1 Laboratory of Marine Biodiversity and Biotechnology, National Institute of Marine Sciences and Technology, BP 59, Monastir, 5000 Tunisia; 2 Biochemistry Laboratory, Research Laboratory in “Nutrition- Functional Food and Vascular Health” Faculty of Medicine, Monastir, 5019 Tunisia; 3 Pharmacology Department, Faculty of Veterinary Medicine, Suez Canal University, Ismailia, 41522 Egypt; 4 Laboratory of Research on Biologically Compatible Compounds, Faculty of Dentisty, Monastir, 5019 Tunisia; 5 Université de Bourgogne Franche-Comté, Laboratoire de Chrono-Environnement, UMR CNRS, Besançon, 6249 France

**Keywords:** Hot spring water, *Leptolyngbya* sp., Antioxidant activities, Phytochemical content, Phenolic profile

## Abstract

**Background:**

In Tunisia, the use of hot spring waters for both health and recreation is a tradition dating back to Roman times. In fact, thermal baths, usually called “Hammam” are recommended as a therapeutic and prophylactic measure against many types of illness and toxicity. While the chemical concentration of thermal water is admittedly associated with its therapeutic effects, the inclusion in spa waters of efficient bioproduct additives produced by photosynthetic microorganisms and that act against oxidative stress may comprise a significant supplementary value for thermal centers. The aim of this study was to investigate the antioxidant potential of the Tunisian thermophilic cyanobacterium *Leptolyngbya* sp. and to determine its phytochemical constituents and phenolic profile.

**Methods:**

BME (Biomass Methanolic Extract), CME (Capsular polysaccharides Methanolic Extract) and RME (Releasing polysaccharides Methanolic Extract) of *Leptolyngbya* sp. were examined for their antioxidant activities by means of DPPH, hydroxyl radical scavenging and ferrous ion chelating assays. Their total phenols, flavonoids, carotenoids, Mycosporine-like amino acids (MAAs) and vitamin C contents, as well as their phenolic profiles were also determined.

**Results:**

BME has the highest content of phenols (139 ± 1.2 mg/g), flavonoids (34.9 ± 0.32 mg CEQ/g), carotenoids (2.03 ± 0.56 mg/g) and vitamin C (15.7 ± 1.55 mg/g), while the highest MAAs content (0.42 ± 0.03 mg/g) was observed in CME. BME presented both the highest DPPH and hydroxyl radical scavenging ability with an IC_50_ of 0.07 and 0.38 mg/ml, respectively. The highest ferrous chelating capacity was detected in CME with an IC_50_ = 0.59 mg/ml. Phenolic profiles revealed the presence of 25 phenolic compounds with the existence of hydroxytyrosol, oleuropein, resveratrol and pinoresinol.

**Conclusion:**

The study demonstrated that the cyanobacterium *Leptolyngbya* sp. possesses abundant natural antioxidant products which may have prophylactic and therapeutic effects on many types of illness and toxicity. The present findings not only explain and reinforce the rationale behind traditional therapeutic practices in Tunisia in the exploitation of the country’s hot springs, but support the addition of *Leptolyngbya* to thermal waters as a means to enhance the value and reputation of the curative nature of Tunisian thermal waters.

## Background

The use of thermal spring waters for health and recreation in Tunisia is a traditional activity dating back to Roman times. This tradition continues today through balneotherapy, also called spa therapy, which is practiced in a Turkish bath also known as a “*Hammam*”, and is recommended as a therapeutic and prophylactic measure against many types of illness and toxicity [1]. In Tunisian spa resorts, as in many countries in the world (Japan, New Zealand, France, Spain, Greece…etc.), the use of hot springs shows similarities. The spa guest can recover by bathing in or drinking thermal water, or by inhaling its vapors [2]. Bathing is mainly recommended for skin care, joint and muscle problems and arthritis. Inhaling is used for the treatment of chronic diseases of the upper and lower airways. Drinking the water is beneficial for some specific diseases. The mechanisms by which broad spectrums of disease are alleviated by spa therapy have not been fully elucidated. [3].

While the chemical concentrations in thermal waters are admittedly associated with their therapeutic effects [4], the inclusion of efficient bioproduct additives produced by photosynthetic organisms and which act against oxidative stress may comprise a significant supplementary value for the increasingly competitive sector of balneotherapy. To accomplish this, these organisms must tolerate: 1) the thermal stress generated in hot thermal spring waters, and 2) an antibiotic additive for the prevention of bacterial proliferation. We hypothesized that the thermophilic microorganisms inhabiting thermal springs-especially cyanobacterial strains-might be likely candidates for bioproduct additives. Indeed, cyanobacteria are photosynthetic and gram-negative, capable of occupying roughly all environments on earth that are visible-light illuminated, with extremophile cyanobacteria thriving in many widely varying habitats such as the Dead Sea, deserts, snow and the outflow of geothermal springs [5, 6]. Their adaptation to extreme conditions is mostly due to the modification of membranes, nucleic acid structure and to the production of efficient protective bioproducts including enzymatic and nonenzymatic antioxidants which combat oxidative stress [7–11] through free radical scavenging that inhibits lipid peroxidation, and to the chelating of metal ions which induce oxidation [7, 12]. For example, evidence is now accumulating as to the links existing between oxidative stress and various diseases including cancer, neurodegenerative disorders, diabetes, cardiovascular diseases, inflammation and rheumatoid arthritis [13–15]. Enzymatic antioxidants include mainly superoxide dismutase (SOD), catalase and glutathione peroxidase, while non enzymatic antioxidants are composed of carotenoids, ascorbic acid, tocopherols, Mycosporine-like amino acids (MAAs) and phenolic compounds [6, 16–18].

The subject of this study is thus the prophylactic and therapeutic potential of Tunisian hot springs in which the thermophilic cyanobacterium *Leptolyngbya* sp. proliferates. Our objective was twofold: 1) to determine the phytochemical constituents, the phenolic profile and the antioxidant activities of the strain’s methanolic extracts, along with both its capsular and releasing polysaccharides, and 2) to explore the possible advantages of the potential use of cyanobacterium in thermal baths in “salus per aquam” (SPA) resorts. The results may have a positive impact on Tunisian thermal tourism activity.

In this study we show for the first time, to the best of our knowledge, the presence of various phenolic compounds including hydroxytyrosol, oleuropein, naphtoresorcinol, catechin, luteolin 7 glucoside, naringenin, flavon, resveratrol and pinoresinol in the cyanobacterium biomass, capsular polysaccharides and releasing polysaccharides.

## Methods

### Reagents

All chemicals, solvents and standards, including phenolic acids were purchased from Sigma-Aldrich Co. Ltd (St. Louis, MO, USA).

### Site description and sample collection

**Fig. 1:**
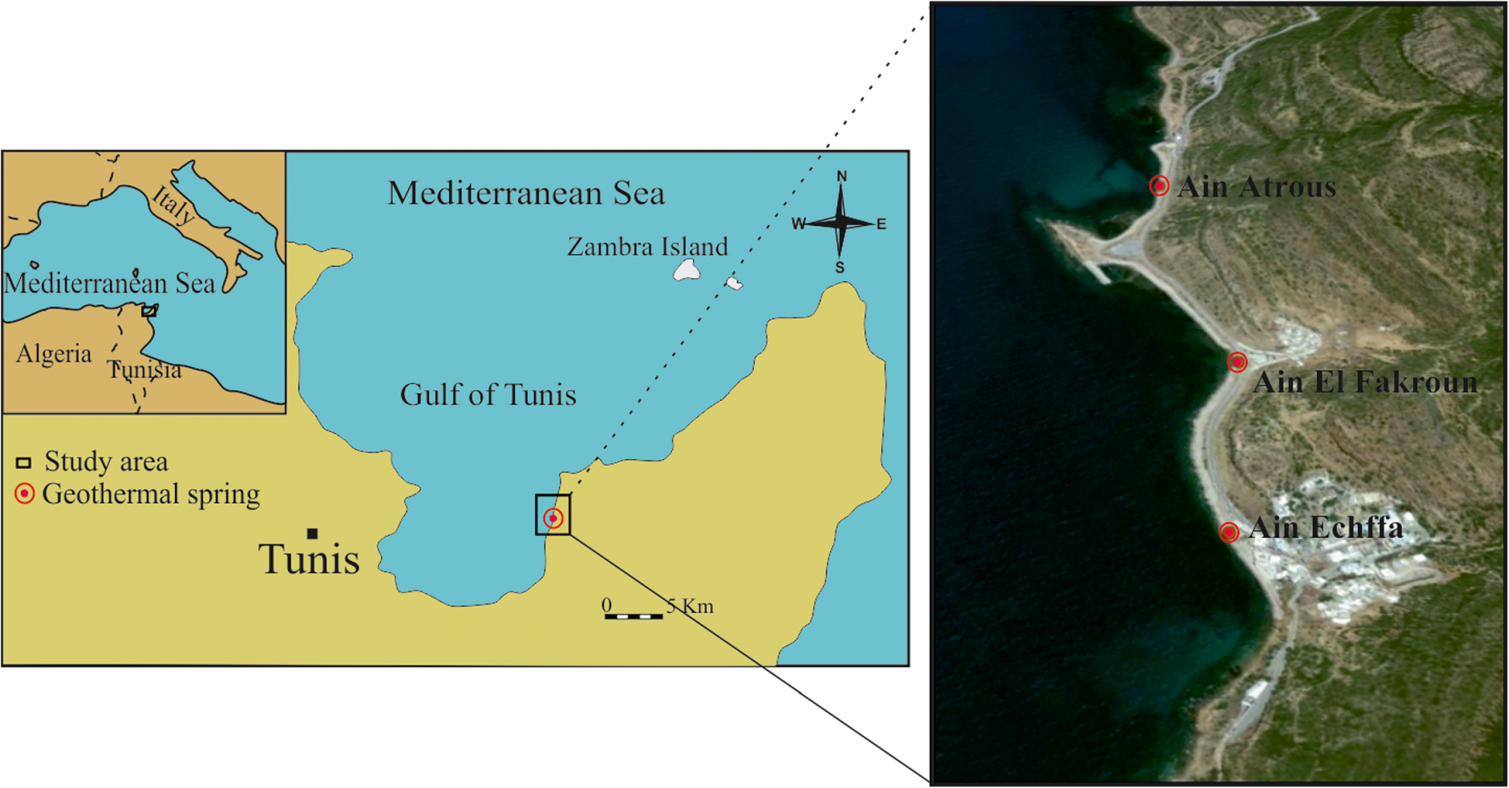
Location of the Aîn Echffa, Aîn El Fakroun and Aîn Atrous thermal springs

Samples were taken from three hot springs: Aîn Echffa, Aîn El Fakroun and Aîn Atrous, in the Korbous region (36 °C 81 ′N, 36 °C 56 ′E) of northern Tunisia (Fig. 1). Microbial mats were collected by scraping submerged rocks using sterile forceps and then stored directly in sterile tubes. Thermal water for cyanobacterial and microalgal cultures was collected in sterile glass, as close as possible to the spring discharge point and added to the sterile tubes containing the microbial mats. The collected samples were treated by filtration, centrifugation and dilution techniques according to standard microbiological protocols [17].

### Strain isolation and culture medium

A dilution and incubation series in 96 well plates under different culture conditions (temperature/light intensity/dark–light cycle) and in different culture media was undertaken with the aim of defining optimal growth conditions for each cyanobacteria and microalgae. Following microscopic examination, the best-growing cultures were selected. *Leptolyngbya* sp. was one of the isolated cyanobacteria strains; preliminary in-lab experiments showed that it presented the highest growth rate in BG11 medium. A monospecific and axenic culture of *Leptolyngbya* sp. was detected by often streaking BG11 agar-agar solid medium preceded by a serial dilution in a BG11 liquid medium [19]. The purified strain was then grown in a batch culture under sterile conditions in a BG11 medium. The initial pH was adjusted to 6.8. Cells were cultured in 20-L sterilized glass bottles of water sparkled with air. Cultures were maintained at 40 °C, in light/dark cycles (16:8) with white fluorescent lamps providing 30 μmol photons m^−2^ s^−1^.

### Microscopic observation and morphological identification

Microscopic investigations of samples were carried out using a Zeiss Axiostar light microscope equipped with differential interference contrast at a magnification of x1000. Images of live and fresh strains were taken using a digital camera (Yashica EZF1027). Strains were drawn after observation by light microscopy in order to illustrate key strain characteristics. The *Leptolyngbya* sp. strain was identified based on morphological criteria, according to the taxonomic keys of [20, 21]. Cell dimensions were measured using a calibrated eyepiece micrometer.

The strain was also observed by means of SEM Type JEOL JSM-5400 scanning microscope after fixation in a phosphate buffer (0.2 M, pH 7.2) containing 2.5% glutaraldehyde and following post fixation in osmium tetroxide (1%) and dehydration in an ethanol series.

### Releasing polysaccharides (RPS) isolation

The RPS were purified according to Trabelsi et al. [22]. A *Leptolyngbya* sp. culture in the stationary phase was centrifuged (4,000 g, 10 min at 4 °C) to obtain a culture filtrate containing both the RPS and the culture medium. A tangential ultra-filtration cell (Millipore, Bedford, MA, USA) and Millipore membranes (30 kDa pore size) were used to concentrate EPS to remove low molecular weight compounds; the RPS were washed three times with deionized water. Finally, the RPS were freeze-dried and lyophilized.

### Capsular polysaccharides (CPS) and biomass isolation

CPS and biomass isolation were conducted following Di Pippoa et al. [23] with a few modifications. *Leptolyngbya* sp. cells and their envelopes (capsular polysaccharides [CPS] were re-suspended in distilled water (1:10), incubated at 60 °C for 60 min and then centrifuged at 10,000 g for 20 min to remove the cells which were then washed three times with deionized water, freeze-dried and lyophilized. The supernatant fluid was ultra-filtrated using a tangential ultra-filtration cell (Millipore, Bedford, MA, USA) and Millipore membranes (30 kDa pore size) as described above (RPS isolation). The purified CPS was freeze-dried and lyophilized.

### Methanol extraction

The methanol extracts of the biomass, CPS and RPS were prepared by mixing each lyophilized compound with pure methanol (ratio 1: 10 g/ ml). Methanol was extracted using an orbital shaker in the dark at 4 °C for 24 h. The mixtures were then filtered through a 0.45 μm syringe filter and concentrated by a rotary evaporator at 50 °C. The three extracts BME (Biomass Methanolic Extract), CME (Capsular polysaccharides Methanolic Extract) and RME (Releasing polysaccharides Methanolic Extract) were stored in the dark at 4 °C until analysis.

### Phytochemical composition

#### Determination of total phenols and flavonoids

Total phenolic contents of BME, CME and RME were analyzed according to Montedoro et al. [24] with slight modifications. An amount of 0.4 ml from each extract and 10 ml of diluted Folin–Ciocalteu reagent were mixed. After 1 min incubation, 8 ml of sodium carbonate (75 g/L) were added, and the mixture incubated for 1 h. Absorbance was measured at 765 nm. Total flavonoid contents in BME, CME and RME were assessed following the method of Zhishen et al. [25]. One ml of each methanolic extract was mixed with 4 ml of distilled water. At t = 0 min, 0.3 ml of NaNO_2_ (5%, w/v) was added. After 5 min, 0.3 ml of (10%, w/v) AlCl_3_ were added. At 6 min, 2 ml of a 1 M solution of NaOH were added. To finish, the total volume was brought up to 10 ml by the immediate addition of 2.4 ml of distilled water. The mixture was then shaken and absorbance at 510 nm was read.

#### Determination of total carotenoids

Total carotenoid content was estimated spectrophotometrically as described by Lichtenthaler and Buschmann [26]. Each methanolic extract was diluted 15–300 times with 90% (v/v) methanol in water and the sample absorbance measured at 470, 652 and 665 nm. Carotenoid content was calculated using the Lichtenthaler equations.

#### Determination of total mycosporine-like amino acids (MAAs)

Mycosporine-like amino acids were quantified by reverse-phase isocratic HPLC [27]. A 50 μl aliquot of each extract was injected into the HPLC at a flow rate of 0.5 ml/min. The mobile phase was 25% water–methanol (v/v) with acetic acid at 0.1% (v/v.) The stationary phase was Phenosphere C_8_ column (5 μm pore size, 4.6 x 250 mm). MAA compounds were detected and quantified using 5 photodiode array channels (310, 320, 330, 334 and 360 nm). Oligosaccharide mycosporine-like amino acid (OS-MAA) peak areas at 310 nm were converted to concentration units by using the extinction coefficient 17 L/g/ cm [28].

#### Determination of vitamin C content

Vitamin C was detected as described by Semary [29] using reverse phase HPLC (C_18_ Column) with a mobile phase of methanol: water (97:3), added under isocratic conditions at a flow rate of 0.5 ml/min and using UV detector at 254 nm. The vitamin C was identified by co-chromatography of valid standards (Sigma). By comparing peaks of the standard samples of both the known and unknown concentrations and relating this to the weight of each extract mass from which the unknown concentration sample was derived, the amount of vitamin C could be calculated.

#### Antioxidant activity

Substrate oxidation took place through a chain reaction implying three different stages: initiation, propagation and termination [30]. We thus tested three methods to evaluate the BME, CME and RME effects on each stage: the DPPH assay (initiation), iron chelating (propagation) and hydroxyl radical scavenging activities (termination).

#### DPPH radical scavenging assay

The DPPH (1,1-dihpenyl-2-picrylhydrazyl) scavenging ability was investigated according to Shimada et al. [31]. One ml of each extract solution in different concentrations (0.01 - 2.0 mg/ml) was added to 3 ml of DPPH ethanol solution (0.004%). Absorbance was determined at 517 nm after 30 min.

#### Ferric chelating ability

To evaluate ferric chelating ability, the contents from the tubes containing different concentrations of each extract (0.01–2.0 mg/ml), 0.2 ml ferrozine (5 mM) and 0.05 ml FeCl_2_ (2 mM) were blended and incubated at room temperature for 10 min. Sample absorbance was measured at 562 nm.

#### Hydroxyl radical scavenging activity assay

Hydroxyl radical scavenging ability was estimated according to Smirnoff and Cumbes [32]. In test tubes, 0.5 ml of each extract solution in different concentrations (0.01–2.0 mg/ml) were added to the mixture of 0.3 ml of orthophenanthroline (5 mmol/L), 0.8 ml of phosphate buffer pH 7.4 (0.75 mol/L), 0.3 ml of FeSO_4_ (7.5 mmol/L) and 0.2 ml of H_2_O_2_ (1%). The reaction mixture was incubated for 60 min at 37 °C and absorbance was measured at 532 nm.

The scavenging ability of DPPH and hydroxyl radical scavenging activity assays, along with the ferric chelating abilities were calculated according to the following equation: scavenging ability/chelating ability (%) = (1 – A_sample_/A_control_) × 100. A_control_: Absorbance without the tested samples (control), A_sample_: Absorbance in presence of the tested samples.

Ascorbic acid (vitamin C) was used as a positive control in both the DPPH radical scavenging assay and the hydroxyl radical scavenging activity assay. EDTA was used as a positive control in the ferric chelating assay.

#### HPLC analysis of phenolic composition

HPLC analysis of phenolic composition in the three *Leptolyngbya* sp. methanolic extracts was performed on a C_18_TechnochromEurosphere 100 analytical column (250 mm × 8 mm) using an HPLC Hewlett Packard system (Waldbronn, Germany) composed of an injector (Cotati, CA, USA, volume 20 μl) an HP-1100 pump and a UV detector (280 nm). Twenty μl of each extract previously passed through a 0.45 μm filter were directly injected into the HPLC. The flow rate was set at 0.5 ml min^−1^. The mobile phases were: (A) Acetonitrile and (B) sulfuric acid/water (2:98). A linear gradient was run from 15% (A) and 85% (B) to 40% (A) and 60% (B) for 12 min; it was changed to 60% (A) and 40% (B) in 2 min; after 4 min it was changed again to 80% (A) and 20% (B); and then to 90% (A) and 10% (B) after 2 min (20 min, total time). After 4 min (out of 24 min) it reached 100% (A) for 4 min. The data were stored and processed using an HPLC Chemstation (Dos Series; Hewlett Packard). The phenolic compounds were determined based on their retention times, and quantified using external standard calibration curves. The results are expressed as mg/g of DW.

#### Statistical analysis

For all experiments results were shown as means ± standard deviation (SD) (*n* = 3). Data were subjected to statistical analysis using the SPSS program, release 11.0 for Windows (SPSS, Chicago, IL, USA). One-way analysis of variance (ANOVA) and then the Duncan multiple range test were used to study the differences between individual means deemed to be significant at *p* < 0.05. EC_50_ values were obtained by interpolation from non-linear regression analysis using Microcal (TM) Origin, version 6.0.

## Results

### Microscopic observation and morphological identification

**Fig. 2:**
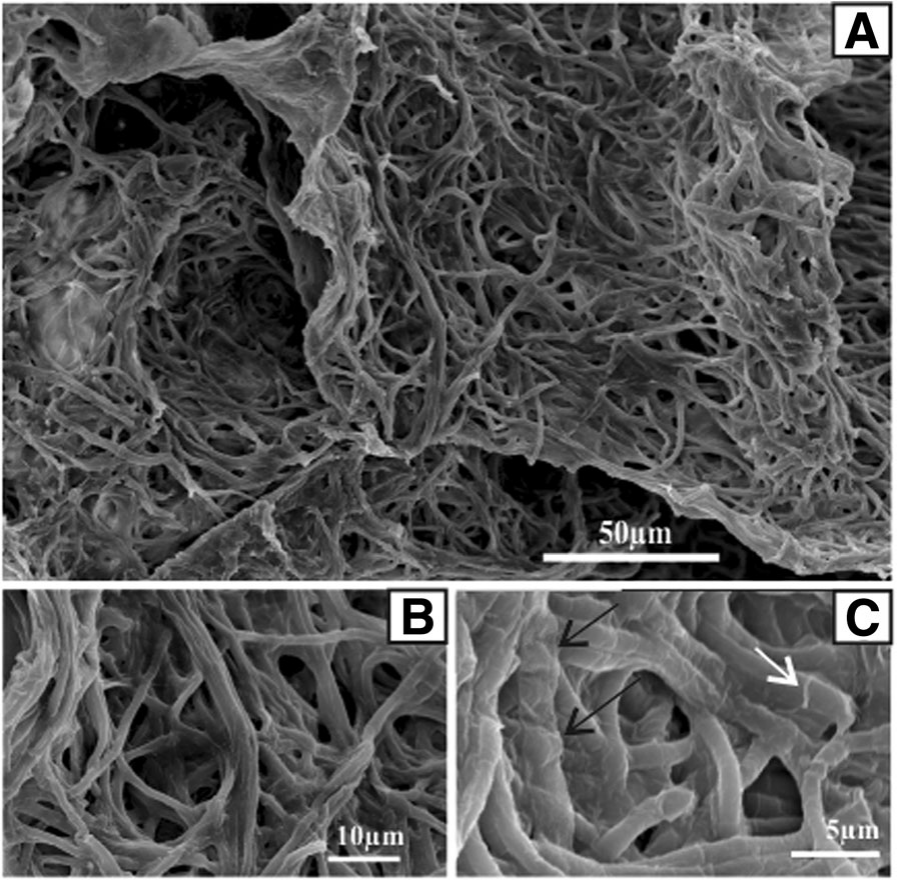
Scanning electron photomicrographs. **a** Global view of mat network, scale bar = 50 μm. **b** Detailed view of fine and cylindrical trichomes (0.7-1.3 μm of diameter), scale bar = 10 μm. **c** Capsular polysaccharides (CPS) at the cell surface are indicated by arrowheads and RPS that have been released are indicated by white arrow, scale bar = 5 μm

The Tunisian thermophilic cyanobacterium *Leptolyngbya* sp. is a phenotypically simple cyanobacterium consisting of a long thin filament (Fig. 2a) surrounded by a transparent sheath which is occasionally open at each end (Fig. 2b). The sheath is thin, transparent and sometimes presents a fibrillary structure (RPS). In some cases the sheath becomes thick and mucilaginous and forms CPS (Fig. 2c). Each cylindrical trichomeis composed of cells which are longer than they are wide. Cells are 2 to 4.5 μm in length and 1 to 2.5 μm in width.

### Phytochemical composition

In this study we evaluated the contents of total phenols (mg GAF/g DW), total flavonoids (mg CEQ/g DW), total carotenoids (mg/g DW), MAAs (mg/g DW) and vitamin C (mg/g DW) for biomass (BME), capsular (CME) and releasing polysaccharide (RME) methanolic extracts of the cyanobacterium *Leptolyngbya* sp.

The results shown in Table 1 demonstrate that there are significant differences for all phytochemical components in the three extracts. In fact, BME has the highest content of phenols (139 ± 1.2 mg/g), flavonoids (34.9 ± 0.32 mg CEQ/g), carotenoids (2.03 ± 0.56 mg/g) and vitamin C (15.7 ± 1.55 mg/g) compared to CME and RME. In contrast, the highest MAAs content (0.42 ± 0.03 mg/g) was observed for CME.

**Table 1:** Concentrations of total phenols, flavonoids, carotenoids, MAAs and vitamin C in the Tunisian thermophilic cyanobacterium *Leptolyngbya*sp

	BME	CME	RME
Phytochemical contents			
Total phenols (mg GAF/g DW)	139 ± 1.2^a^	34.2 ± 0.96^b^	23.2 ± 0.11^c^
Total flavonoids (mg CEQ/g DW)	34.9 ± 0.32^a^	18.6 ± 0.41^b^	15.33 ± 0.58^c^
Total carotenoids (mg/g DW)	2.03 ± 0.56	nd	nd
Total MAAs (mg/g DW)	nd	0.42 ± 0.03^a^	0.23 ± 0.02^b^
Total vitamin C (mg/g DW)	15.7 ± 1.55	nd	nd

*GAF* gallic acid equivalents, *CEQ* catechin equivalents, *MAAs* mycosporine-like amino acid. Values are means ± SD (*n* = 3): means in the same rows representing different letters are significantly different at *p* < 0.05; “nd” = not detected

Methanol Extracts: Biomass (BME), Capsular polysaccharides (CME) and Releasing polysaccharides (RME)

### Antioxidant activity

**Fig. 3:**
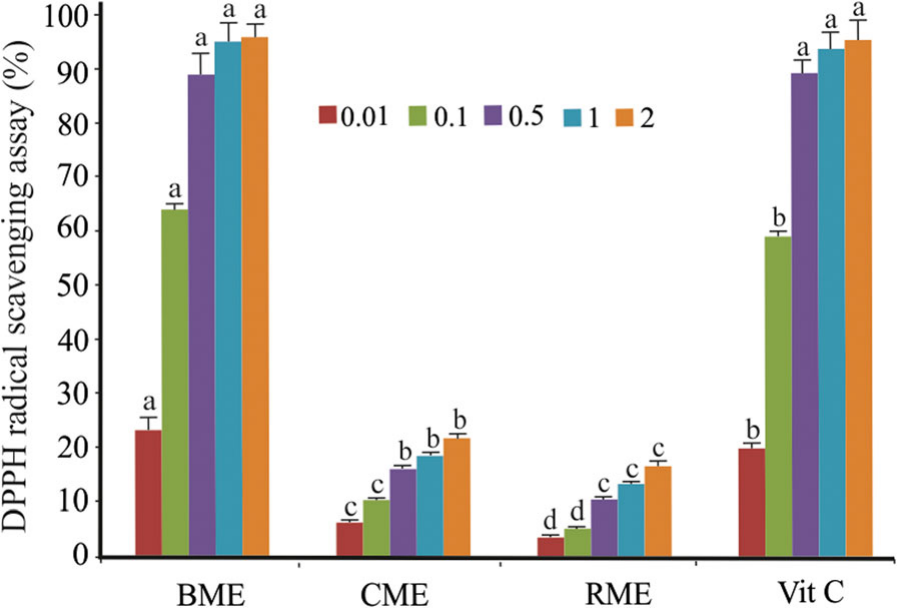
DPPH radical scavenging activity of the BME, CME and RME in different concentrations. Values are means ± SD (*n* = 3). Different small letters within the histogram are significantly different (*p* < 0.05) with respect to the extract concentration and the control according to the Duncan test

**Fig. 4:**
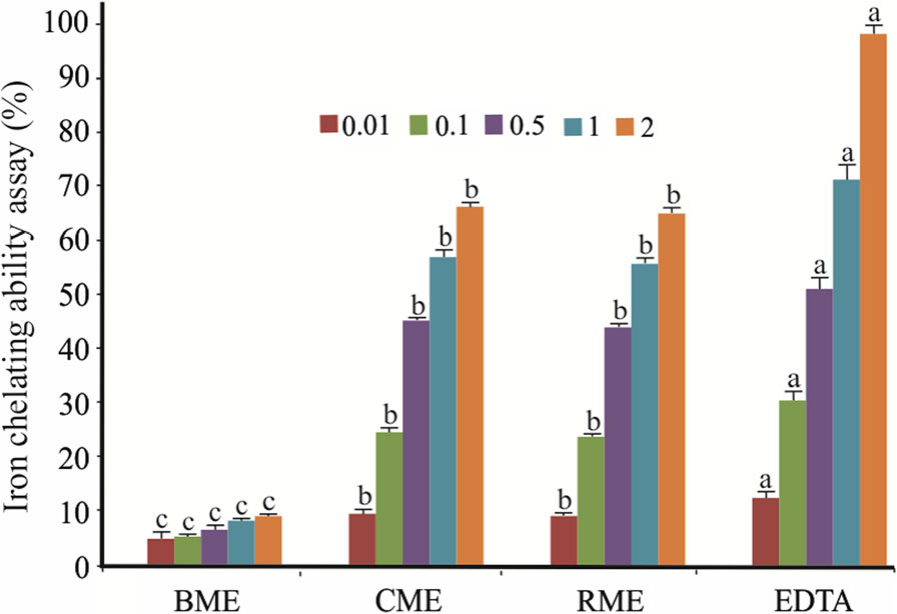
Chelating ability of BME, CME and RME in different concentrations. Values are means ± SD (*n* = 3). Different small letters within the histogram are significantly different (*p* < 0.05) with respect to the extract concentration and the control according to the Duncan test

**Fig. 5:**
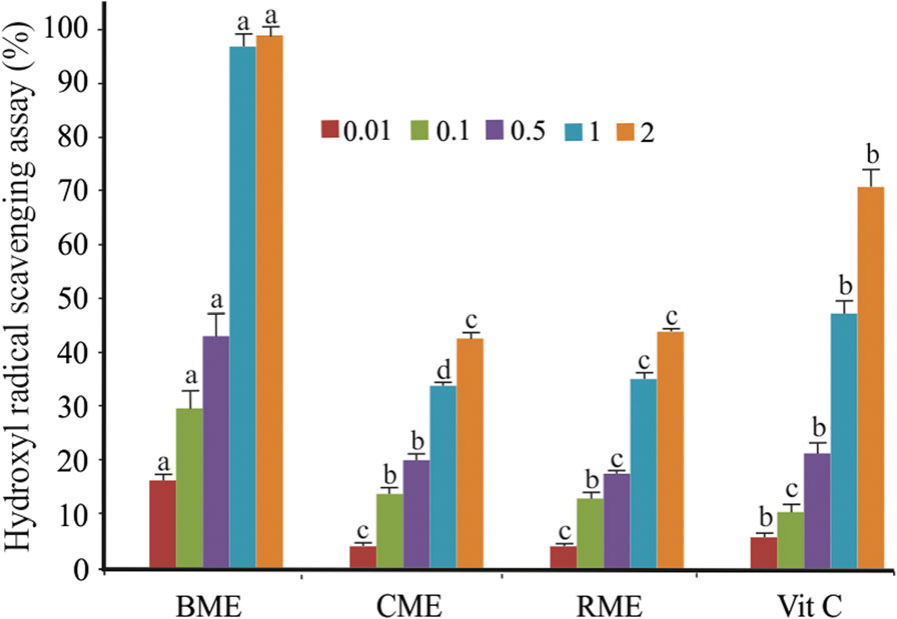
Hydroxyl radical scavenging activity of BME, CME and RME in different concentrations. Values are means ± SD (*n* = 3). Different small letters within the histogram are significantly different (*p* < 0.05) with respect to the extract concentration and the control according to the Duncan test

The results of DPPH, ferrous ion chelating and hydroxyl radical scavenging assays are depicted in Figs. 3, 4 and 5, respectively.

The results of the DPPH assay obviously showed that there were significant differences (*p* < 0.05) in terms of their scavenging abilities present among BME, CME and RME. Among the three extracts, BME displayed the highest radical scavenging activity (98.2 ± 2.4%), with the lowest (18.3 ± 0.6%) found in RME. The *Leptolyngbya* sp. BME presented a high radical scavenging ability with an IC_50_ equal to 0.07 mg/ml (Table 2), a capacity apparently dose-dependent. When compared to vitamin C used in the same concentrations and under the same experimental conditions, no significant differences were observed. In fact, for both BME and vitamin C, the maximum scavenging ability reached when using 2 mg/ml was 98.2 ± 2.4% and 97.3 ± 3%, respectively.

**Table 2:** EC_50_ values of Tunisian thermophilic cyanobacterium *Leptolyngbya* sp

	BME	CME	RME	Vit C/EDTA
DPPH radicals scavenging assay	0.07	nd	nd	0.07
Ferric Chelating ability	nd	0.59	0.63	0.59
Hydroxyl radical scavenging assay	0.38	nd	nd	1.17

EC_50_ values were obtained by interpolation from non-linear regression analysis; *nd* not detected

Methanol Extracts: Biomass (BME), Capsular polysaccharides (CME) and Releasing polysaccharides (RME)

The results of the ferrous ion chelating assays revealed that CME presented the highest ferric chelating capacity with an IC_50_ equal to 0.59 mg/ml (Table 2). When compared to EDTA, CME showed a moderate chelating ability. In fact, the maximum chelating capacity was only 65.7 ± 0.8%, a value reached using 2 mg/ml of CME. For the same concentrations, the iron chelating activity of the EDTA was 1.45 times higher than CME activity (*p* < 0.05).

The results of the hydroxyl radical scavenging assays of the three extracts displayed that BME presented the highest inhibition value (98.2 ± 3.1%) and exhibited a significant decrease in a concentration-dependent manner of the hydroxyl radical. Its maximal inhibition value was observed for 2 mg/ml and its IC_50_ value was 0.38 mg/ml.

### HPLC analysis of phenolic composition

Shown in Table 3, HPLC analysis of *Leptolyngbya* sp. methanolic extracts (BME, CME and RME) reveals the presence of 25 phenolic compounds:

**Table 3:** Phenolic profiles of Tunisian thermophilic *Leptolyngbya* sp: BME, CME and RME (in mg/g DW)

	BME	CME	RME
Gallic	14.2 ± 0.1^a^	0.4 ± 0.02^b^	0.3 ± 0.01^c^
Hydroxytyrosol	4.0 ± 0.1^a^	0.1 ± 0.02^b^	0.1 ± 0.02^b^
Protocatechuic	0.5 ± 0.1^c^	1.3 ± 0.01^a^	1.1 ± 0.03^b^
Vanillic	6.0 ± 0.02^a^	0.8 ± 0.03^b^	0.5 ± 0.02^c^
Isovanillic	0.2 ± 0.01	nd	nd
3-hydroxybenzoic	1.1 ± 0.1^a^	nd	0.1 ± 0.02^b^
4-Hydroxybenzoic	0.5 ± 0.02^a^	0.1 ± 0.01^b^	0.1 ± 0.01^b^
Catechol	nd	0.1 ± 0.01^a^	0.1 ± 0.01^a^
Resorcinol	0.6 ± 0.01^a^	0.3 ± 0.01^c^	0.4 ± 0.02^b^
Naphtoresorcinol	1.4 ± 0.01^a^	0.6 ± 0.01^c^	0.8 ± 0.02^b^
Syringic	1.7 ± 0.1	nd	nd
Oleuropein	2.0 ± 0.1^a^	0.2 ± 0.01^b^	0.2 ± 0.01^b^
Ʃ HBA	32.2 ± 0.05^a^	3.9 ± 0.02^b^	3.7 ± 0.02^c^
Chlorogenic	0.1 ± 0.01	nd	nd
P coumaric	1.1 ± 0.1^a^	0.6 ± 0.01^b^	0.5 ± 0.02^c^
M coumaric	0.8 ± 0.02^a^	0.2 ± 0.01^c^	0.3 ± 0.01^b^
Ferulic	9.3 ± 0.03^a^	0.1 ± 0.01^c^	0,2 ± 0.02^b^
Rosameric	0.7 ± 0.01^a^	0.6 ± 0.01^b^	0.4 ± 0.02^c^
Ʃ HCA	12 ± 0.03^a^	1.5 ± 0.01^b^	1.4 ± 0.02^c^
Catechin	2.6 ± 0.2^a^	nd	0.1 ± 0.02^b^
Luteolin 7 glucoside	4.5 ± 0.03^a^	0.4 ± 0.01^b^	0.3 ± 0.02^c^
Apigenin	0.4 ± 0.02^a^	0.2 ± 0.01^b^	0.2 ± 0.01^b^
Apigenin 7 glucoside	0.4 ± 0.01^b^	0.5 ± 0.02^a^	0.5 ± 0.02^a^
Naringenin	4.1 ± 0.01^a^	1.2 ± 0.2^b^	1.0 ± 0.1^c^
Luteolin	0.2 ± 0.01^b^	0.4 ± 0.01^a^	0.2 ± 0.01^b^
Flavon	0.7 ± 0.02^b^	0.9 ± 0.02^a^	0.5 ± 0.03^c^
Ʃ Flavonoids	12.9 ± 0.07^a^	3.6 ± 0.04^b^	2.8 ± 0.03^c^
Resveratrol	0.2 ± 0.01^c^	0.4 ± 0.01^a^	0.3 ± 0.02^b^
Pinoresinol	1.3 ± 0.01^b^	2.2 ± 0.1^a^	0.5 ± 0.02^c^
Ʃ Phenolic	58.6 ± 0.03^a^	11.6 ± 0.04^b^	8.7 ± 0.02^c^

Values are means ± SD (*n* = 3): means in the same rows representing different letters are significantly different (*p* < 0.05); HBA: Hydroxybenzoic acids; HCA: Hydroxycinnamic acids; “nd” = not detected

- twelve hydroxybenzoic acids (HBA): gallic, hydroxytyrosol, protocatechuic, vanillic, isovanillic, 3-HBA, 4-HBA, catechol, resorcinol, naphtoresorcinol, syringic, and oleuropein;
- five hydroxycinnamic acids (HCA): chlorogenic, p-coumaric, m-coumaric, ferulic, and rosameric acids;
- seven flavonoids: catechin, luteolin-7-glucoside, apigenin, apigenin-7-glucoside,naringenin, luteolin, and flavon;
- one stilbene (resveratrol); and
- one lignane (pinoresinol).

As illustrated in Table 3, HBA (32.2 ± 0.05 –3.9 ± 0.02 mg/g) were the most preponderant phenolic compounds observed in BME, CME, and RME, followed by flavonoids (12.9 ± 0.07 –3.6 ± 0.04 mg/g) and HCA (12 ± 0.03–1.5 ± 0.01 mg/g). The highest HBA, HCA and flavonoid levels were observed in BME whereas the lowest were obtained in RME. The levels of the main phenolic compounds were extract-dependent. Among the HBA, gallic acid, followed by vanillic acid were the most abundant in BME (14.2 ± 0.1 mg/g - 6.0 ± 0.02 respectively) (*p* < 0.05), whereas protocatechic was the most predominant in CME and RME (1.3 ± 0.01 - 1.1 ± 0.03 mg/g, respectively). Among the HCA, ferulic acid was the most predominant compound in BME (9.3 ± 0.03 mg/g). P-coumaric acid was the main HCA compound in CME and RME (0.6 ± 0.01 and 0.5 ± 0.02 mg/g, respectively). The main flavonoids were luteolin 7 glucosides (4.5 ± 0.03 mg/g) and naringenin in BME, while only naringenin in CME (1.2 ± 0.2 mg/g) and RME (1.0 ± 0.1 mg/g). For the stilbene resveratrol and the lignane pinoresinol, the highest amounts (0.4 ± 0.01and 2.2 ± 0.1 mg/g, respectively) were recorded in CME.

## Discussion

This study confirmed the presence of diverse phytochemicals and antioxidant activities in the methanolic extracts of the biomass (BME), the capsular (CME) and the releasing polysaccharides (RME) of the Tunisian thermophilic cyanobacterium *Leptolyngbya* sp.

The *Leptolyngbya* BME presented the highest concentrations of phenols, flavonoids and vitamin C, the highest scavenging ability of DPPH free radical and the highest hydroxyl radical scavenging ability. The concentrations of phenols, flavonoids and vitamin C were found in the *Leptolyngbya* sp. BME and were higher than those reported by Ijaz and Hasnain [33] for the genus *Leptolyngbya*, and by Rai and Rajashekhar [34] for other cyanobacteria strains (*Phormidium corium*, *Oscillatoria fremyii, Spirulina major…*)*.* These differences may be attributed either to the cyanobacterial strains and their environmental origins or to the extraction methods and solvents used. Definitely, the high amount of phenols, flavonoids and vitamin C in our case may be considered as a way to avoid oxidative stress induced by the high temperature levels in thermal spring water [6]. Furthermore, methanol is the most commonly used solvent for phenolic extraction due to its high polarity and its wide solubility properties.

The high level of DPPH radical scavenging activity of BME is mostly attributed to its high content in phenolic acids (particularly gallic, ferulic and vanillic) and in flavonoids (mainly luteolin 7 glucoside and naringenin). Phenolic acids and flavonoids are potent free radical scavengers and so possess antioxidative properties [35–37]. The high-level accumulation of these phenolic compounds in the biomass of the thermophilic cyanobacteria *Leptolyngbya* sp. may be an important mechanism for self-protection when under stressful conditions. This strategy has been well described by Dhananjaya et al. [38].

Hydroxyl radical is one of the most reactive oxygen species in the body. It severely damages proximate bio-molecules (DNA, protein) resulting in mutagenesis, carcinogenesis and cytotoxicity [39]. Removal of the hydroxyl radical from living organisms thus protects them from different illness and diseases. The results of the hydroxyl radical scavenging ability demonstrated that BME was the most powerful with an IC_50_ = 0.38 mg/ml and exhibited a significant decrease in a concentration-dependent manner of the hydroxyl radical. This result is in accordance with an earlier published paper [40] and leads us to believe that BME may be considered to be a potent quencher of the hydroxyl radical and that the Tunisian thermophilic cyanobacterium *Leptolyngbya* sp. might help the human body to prevent oxidative damage. Moreover, the presence of hydroxytyrosol and oleuropein in BME, well known for its hydroxyl radical scavenging capacity [41], must be reported.

In this study, we have also demonstrated that the highest content of MAAs was observed for CME which means that *Leptolyngbya* sp. has the ability to accumulate MAAs in its capsular polysaccharides. The existence of MAAs in cyanobacteria has been reported since 1969 by several authors [42–46]. However, despite this evidence the exact location of MAAs in cyanobacteria is not well known, except in certain cyanobacterial strains (*Nostoc commune*, *Arthrospira platensis and Microcoleus* sp.) in which they have been shown to be actively secreted and cumulated extracellularly [22, 28]. These observations show good agreement with our results.

The DPPH radical scavenging capacity of CME and RME was moderate and did not exceed 22.3 ± 1.1%. According to Hajimahmoodi et al. [47], the aqueous extract of *Chlorella vulgaris* extracellular polysaccharides showed an activity in the area of 109.02 ± 8.25% of radical scavenging in the DPPH assay, a result in stark contrast to our data, the difference being essentially attributed to the biochemical composition of the extracts for each extracellular polysaccharide. Indeed, the EPS aqueous extract of *Chlorella vulgaris* was rich in phenolic compounds whereas the capsular and releasing polysaccharides of the *Leptolygbya* sp. methanol extracts were rich in MAAs. When compared with BME, CME and RME (the EPS aqueous extract of *Chlorella vulgaris*) presented high ferrous ion chelating ability. This inevitably led us to predict that CME and RME contained polysaccharides enabling iron chelating ability. This prediction was verified in our laboratory (data not shown). In fact, the compound’s chelating ability is described by Melo-Silveira et al. [30] as: “the formation of bonds between two or more binding sites within the same molecule and a single central atom”. This specificity was mainly observed in organic substances such as polysaccharides, which have the ability to bind to metal atoms from chelate [48]. The hydroxyl radical scavenging capacity of CME and RME was considered moderate compared to BME, but promising compared to other cyanobacterial extracts [49].

In HPLC analysis, 25 compounds were identified in BME while 21 were identified in CME and 23 in RME. According to numerous studies the most predominant phenolic compounds in cyanobacteria are gallic acid, vanillic acid, syringic acid, ferulic acid, chlorogenic acid, 3.4-dihydroxybenzoic acid, protocatechuic acid, caffeic acid, coumaric acids and rutin [33, 50–52]. Only slight variability is observed compared to our data. This difference is essentially attributed to the presence of nine other phenolic compounds: hydroxytyrosol, oleuropein, naphtoresorcinol, catechin, luteolin 7 glucoside, naringenin, flavon, resveratrol and pinoresinol, and to the absence of caffeic acid and rutin. The absence of some phenolic compounds may be attributed to auto-oxidation, and especially to enzymatic oxidation by peroxidase and polyphenol oxidase [53]. Dhananjaya et al. [38] demonstrated that rutin and caffeic acid were mainly observed for cyanobacteria under salt stress but not under thermal stress. The existence of hydroxytyrosol and oleuropein—the major polyphenols in olives—in the *Leptolyngbya* sp. BME at 4**.**0 ± 0.1 and 2.0 ± 0.1 mg/g DW, respectively, must be emphasized. In fact, hydroxytyrosol prevents bone loss [54], whereas oleuropein is considered as a medicinal compound with diverse biological properties such as antidiabetic, anti-cancer and anti-atherosclerotic properties [55]. Furthermore, special attention must be paid to stilbene and resveratrol, also observed in the three methanol extracts of the Tunisian thermophilic cyanobacterium *Leptolyngbya* sp. In fact, resveratrol has been reported to prevent atherosclerosis and to be useful in treating some chronic diseases such as neurodegenerative disorders and diabetes mellitus [56].

Bathing in hot springs increases body temperature, which increases blood flow, resulting in the increased absorption capability of the intestines [57]. It has been also reported that bathing below 40 °C stimulates parasympathetic activity and activates gastroenteric digestive functions [58]. These findings lead us to hypothesize that bathing, along with thermal water consumption, may offer the appropriate level of absorption of the *Leptolyngbya* phenolic compounds for therapeutic effect. Furthermore, the human epidermis barrier function is important to transdermal delivery of drugs, and its permeability to many phenolic compounds was proved by Roberts et al. [59]. Phenolic compounds are widely used in topical preparations for their local anesthetic, antipruritic or antibacterial properties; they are generally applied to the skin either as preservatives or to obtain a local effect [59]. Immersion in hot spring water and application of jet-water opens the skin pores and can facilitate the penetration of the *Leptolyngbya* sp. antioxidant compounds.

## Conclusion

This study demonstrates that the Tunisian thermophilic cyanobacterium *Leptolyngbya* sp. may constitute a potential source of natural antioxidant products such as vitamin C, phenolic compounds, flavonoids and mycosporine-like amino acids (MAAs). The strain’s phenolic profiles also reveal the presence of 25 phenolic compounds, with the existence of hydroxytyrosol, oleuropein, and resveratrol polyphenols well-known for their therapeutic and disease-preventive applications. The present findings not only explain and reinforce the rationale behind Tunisia’s traditional therapeutic practices in the exploitation of the country’s hot springs, but also support the addition of *Leptolyngbya* to thermal waters as a means of enhancing the value and reputation of the curative nature of Tunisian thermal waters.

With approximately 150,000 patients per year and nearly 450 spas, Tunisia is today the second-ranking destination in the world after France for the treatment of certain diseases through balneotherapy and hydrotherapy [60]. This activity should be preserved and further developed in the context of Tunisia’s expanding economy, ultimately fostering a symbiosis between health and recreation in thermal tourism [61].

## Abbreviations

BME: Biomass methanolic extract
CEQ: Catechin equivalents
CME: Capsular polysaccharides methanolic extract
CPS: Capsular polysaccharides
GAF: Gallic acid equivalents
HBA: Hydroxybenzoic acids
HCA: Hydroxycinnamic acids
MAAs: Mycosporine-like amino acids
RME: Releasing polysaccharides methanolic extract
RPS: Releasing polysaccharides
SEM: Scanning electron microscope
